# Shifts in structural connectome organization in the limbic and sensory systems of patients with episodic migraine

**DOI:** 10.1186/s10194-024-01806-2

**Published:** 2024-06-11

**Authors:** Eunchan Noh, Jong Young Namgung, Yeongjun Park, Yurim Jang, Mi Ji Lee, Bo-yong Park

**Affiliations:** 1 College of Medicine, Inha University, Incheon, Republic of Korea; 2 Department of Data Science, Inha University, Incheon, Republic of Korea; 3 Department of Electrical and Computer Engineering, Sungkyunkwan University, Suwon, Republic of Korea; 4 Department of Statistics and Data Science, Inha University, Incheon, Republic of Korea; 5 Department of Neurology, Seoul National University Hospital, Seoul National University College of Medicine, Seoul, Republic of Korea; 6 Center for Neuroscience Imaging Research, Institute for Basic Science, Suwon, Republic of Korea

**Keywords:** Episodic migraine, Limbic system, Manifold eccentricity, Structural connectivity, Tractography

## Abstract

Migraine is a complex neurological condition characterized by recurrent headaches, which is often accompanied by various neurological symptoms. Magnetic resonance imaging (MRI) is a powerful tool for investigating whole-brain connectivity patterns; however, systematic assessment of structural connectome organization has rarely been performed. In the present study, we aimed to examine the changes in structural connectivity in patients with episodic migraines using diffusion MRI. First, we computed structural connectivity using diffusion MRI tractography, after which we applied dimensionality reduction techniques to the structural connectivity and generated three low-dimensional eigenvectors. We subsequently calculated the manifold eccentricity, defined as the Euclidean distance between each data point and the center of the data in the manifold space. We then compared the manifold eccentricity between patients with migraines and healthy controls, revealing significant between-group differences in the orbitofrontal cortex, temporal pole, and sensory/motor regions. Between-group differences in subcortico-cortical connectivity further revealed significant changes in the amygdala, accumbens, and caudate nuclei. Finally, supervised machine learning effectively classified patients with migraines and healthy controls using cortical and subcortical structural connectivity features, highlighting the importance of the orbitofrontal and sensory cortices, in addition to the caudate, in distinguishing between the groups. Our findings confirmed that episodic migraine is related to the structural connectome changes in the limbic and sensory systems, suggesting its potential utility as a diagnostic marker for migraine.

**Supplementary Information:**

The online version contains supplementary material available at 10.1186/s10194-024-01806-2.

## Introduction

Migraine is a neurological disorder characterized by recurrent headaches accompanied by nausea, vomiting, and an increased sensitivity to sound or light [1]. Magnetic resonance imaging (MRI) is widely used as a technique to uncover the neurological underpinnings of migraines [2]. In particular, structural connectivity, defined using diffusion tractography, has previously been adopted to assess changes in inter-regional relationships in tract strengths between different brain regions, beyond the simple investigation of regional abnormalities in patients with migraine [3, 4]. By calculating graph-theoretical parameters such as nodal centrality and efficiency measures, researchers have observed changes in the tract strengths in pain-processing-related brain regions, particularly in the sensory and limbic networks, which were found to be associated with prolonged disease duration and higher pain severity [3, 4].

Several challenges have arisen in these studies. Indeed, previous studies of structural connectivity have reported varying results [3–6]. For example, one study found changes in the structural connectome hubs in the limbic and emotional networks that were not related to disease severity [3]. In contrast, another study observed network differences in the occipital, temporal, and parietal lobes associated with disease severity and duration [4]. This inconsistency may be due to methodological issues in diffusion tractography, such as the choice of features for constructing structural connectivity (e.g., streamline count, density, or cross-section) and tractography methods (e.g., deterministic or probabilistic). As such, methods that can extract major information from structural connectivity data without considering the information that may vary across methodological choices must be considered.

Manifold learning, also called gradient analysis, is one technique that can be used to explore connectome organization [7–10]. The essence of this analytical method lies in dimensionality reduction, which reduces high-dimensional connectome data to multiple low-dimensional eigenvectors (i.e., gradients) [8, 10], allowing for an effective explanation of the entire dataset using a small set of gradients, while retaining sufficient information [11–13]. The reliability of the gradient technique has previously been demonstrated for several neurological disorders [11–14]. For example, our previous study successfully revealed alterations in functional connectome organization in patients with migraines using functional MRI-based gradient analysis [14]. The only limitation of gradient analysis lies in the interpretation of the results, as only multivariate analysis can be used to assess between-group differences across multiple gradients. Thus, the directionality of association cannot be obtained from univariate analysis.

In the present study, we opted to perform gradient analysis to investigate whole-brain structural connectome changes in patients with episodic migraine. Specifically, we used “manifold eccentricity,” which consolidates multiple gradients into a single vector [15]. This enabled us to assess the directionality of the statistics by quantifying the expansion and contraction of the data points in the manifold space. We further hypothesized that patients with migraine would show changes in manifold eccentricity compared to healthy controls.

## Methods

### Study participants

Patients with episodic migraines were recruited from an academic headache clinic between October 2020 and November 2021. The inclusion criteria for patients were: (1) age 18–50 years; (2) not taking preventive medications; and (3) premenopausal status in female patients. The exclusion criteria included: (1) chronic migraine, medication-overuse headaches, chronic pain disorders other than migraine, and psychiatric disorders such as bipolar affective disorder or schizophrenia; (2) contraindications for 3T MRI, including use of a tissue expander, pacemaker, non-detachable metal objects, orthodontic devices, or electrical leads or implants in the body; (3) pregnancy; (4) claustrophobia requiring sedation during MR scanning; (5) inability to report their headache or complete the headache diary due to cognitive decline; and (6) disagreement with the study procedures. Migraine was diagnosed according to the ICHD-3 criteria by a single investigator (MJL), a neurologist specializing in headaches. This study was approved by the Samsung Medical Center Institutional Review Board, and all participants provided written informed consent to participate. This study was part of an ongoing longitudinal project registered at ClinicalTrials.gov (NCT03487978).

### MRI data acquisition

T1-weighted structural and diffusion MRI data were obtained using a Philips Ingenia 3T scanner (Philips, Amsterdam, Netherlands). T1-weighted data were acquired using a turbo field echo sequence in the sagittal plane (repetition time [TR] = 8.1 ms; echo time [TE] = 3.7 ms; field of view [FOV] = 256 × 256 mm^2^; voxel size = 1 mm isotropic; and number of slices = 180). Diffusion MRI data were acquired using a spin-echo echo-planar imaging sequence in the axial plane (TR = 7,062 ms; TE = 91 ms; FOV = 220 × 220 mm^2^; voxel size = 1.719 × 1.719 × 3 mm^3^; number of slices = 47; b-value = 1,000 s/mm^2^; number of diffusion directions = 64; and number of b0 images = 1). T1-weighted MRI scanning was performed for 3 min 20 s, and diffusion MRI for 7 min 50 s.

### MRI data preprocessing

The T1-weighted MRI data were preprocessed using the fusion of neuroimaging preprocessing (FuNP) surface-based pipeline [16], which included gradient nonlinearity correction, non-brain tissue removal, and intensity normalization. The cortical surfaces were generated using FreeSurfer v7.1.1 (Boston, MA, USA) [17] by following the boundaries between the white and pial surfaces [18–20]. The mid-thickness surface was generated by averaging the white and pial surfaces, and was used to generate an inflated surface. Diffusion MRI data were preprocessed using MRtrix3 v3.0.2 [21], including denoising, Gibbs ringing artifact removal, susceptibility distortion correction, head motion correction, and eddy current correction. Anatomically-constrained tractography was further performed using different tissue types of the cortical and subcortical gray matter, white matter, and cerebrospinal fluid, as defined using T1-weighted MRI [22]. T1-weighted and diffusion MRI data were co-registered using FSL v6.0 [23], and different tissue types were transformed into the native diffusion MRI space. After estimating the single-shell response functions [24], constrained spherical deconvolution and intensity normalization were subsequently performed [25]. A tractogram was further generated using a probabilistic approach with 40 million streamlines [26, 27]. Options were set with a maximum tract length of 250 and a fractional anisotropy cutoff of 0.06. Spherical deconvolution-informed filtering of tractograms (SIFT2) was further applied to reconstruct the whole-brain streamlines weighted by cross-section multipliers [28]. The structural connectivity matrix was constructed by mapping the reconstructed cross-section streamlines onto a sub-parcellation of the Desikan–Killiany atlas with 300 parcels [29]. The sub-parcellation of the Desikan–Killiany atlas was defined in MICAPIPE (https://github.com/MICA-MNI/micapipe/tree/master/parcellations*)* [30], which subdivided the FreeSurfer segmentation into several sub-regions.

### Structural connectivity gradient generation

We generated low-dimensional representations of structural connectivity (i.e., gradients) by applying manifold learning, a nonlinear dimensionality reduction technique [10]. The application of manifold learning to high-dimensional data allows the generation of multiple low-dimensional eigenvectors to construct a newly defined low-dimensional space (i.e., a manifold space). First, we constructed a group representative matrix computed using distance-dependent thresholding to preserve long-range connections [31]. Using the BrainSpace toolbox (https://github.com/MICA-MNI/BrainSpace [10]), we then applied diffusion map embedding, a robust and computationally efficient nonlinear dimensionality reduction technique [32, 33], to the group-representative structural connectivity matrix. Individual gradients were estimated by applying diffusion map embedding to the individual structural connectivity matrix and were aligned to group representative gradients using Procrustes alignment [10, 34].

### Manifold eccentricity and between-group differences

We subsequently generated three structural connectivity gradients that sufficiently explained total connectivity information and showed biologically interpretable axes [15, 35, 36]. Multiple gradients were further summarized into a single feature termed manifold eccentricity, defined as the Euclidean distance between each data point and the center of the data in the manifold space [15]. Differences in manifold eccentricity between patients with migraines and healthy controls were assessed using non-parametric permutation tests. The subject indices were randomly shuffled, and an analysis of covariance (ANCOVA) was performed by controlling for age and sex. This process was repeated 10,000 times. A null distribution of the between-group differences was constructed, and the p-value was calculated by dividing the number of absolute permuted statistical values larger than absolute value of the real statistic by the number of permutations. Multiple comparisons across brain regions were corrected using a false discovery rate (FDR) < 0.05 [37]. To quantify the between-group difference effects on manifold eccentricity according to brain networks, we further summarized the statistical values according to seven intrinsic functional communities, including the visual, somatomotor, dorsal attention, ventral attention, limbic, frontoparietal, and default mode cortices [38], and cortical hierarchical levels, including idiotypic, unimodal association, heteromodal association, and paralimbic areas [39].

### Changes in subcortical structures

In addition to investigating the changes in cortical manifold eccentricity, we further assessed the between-group differences in subcortical structural connectivity by analyzing the degree of subcortico-cortical structural connectivity, defined as the sum of edge weights connecting each subcortical region to all cortical regions. The subcortical structures of the thalamus, caudate, putamen, pallidum, hippocampus, amygdala, and accumbens were defined from the T1-weighted data using FSL FIRST [40]. We further conducted 10,000 permutation tests, and multiple comparisons across the subcortical structures were corrected using an FDR threshold of < 0.05.

### Classification between patients with migraine and healthy controls

To validate the above features, we adopted supervised machine learning to classify patients with migraines and healthy controls using the cortical manifold eccentricity and degree values of subcortico-cortical connectivity. Specifically, following the regression of age and sex from the features, we applied the least absolute shrinkage and selection operator (LASSO) method to select the imaging features [41], and entered the selected features into a linear regression model. We further performed the classification task using a five-fold cross-validation framework by dividing the data into training (4/5 partitions) and testing (1/5 partitions) datasets. The procedure was repeated 100 times using different training and testing datasets to avoid subject selection bias. Classification performance was assessed using precision, recall, and area under the receiver operating characteristic (ROC) curves (AUC), and the mean scores with standard deviation (SD) across 100 repetitions were reported.

### Sensitivity analysis

#### A) Excluding patients with migraine with aura

To evaluate the impact of migraine with or without aura on between-group differences between patients with migraine and healthy controls, we further assessed the differences in the manifold eccentricity and degree values of subcortico-cortical connectivity after excluding patients with migraine who had an aura (*n* = 7).

#### B) Different parcellations

To assess the robustness of the findings across different spatial scales, we repeated the analyses using a sub-parcellation of the Desikan–Killiany atlas with 200 and 400 parcels [29]. We further performed the same analyses using the Schaefer atlas with 300 parcels to represent a functional parcellation scheme [42].

#### C) Different migraine phases

We obtained the participants’ headache status within ± 2 days of MRI scanning. Patients were considered ictal if they had headaches of any intensity on the day of scanning, interictal if they were headache-free on ± 2 days of scanning, and peri-ictal if they were headache-free on the day of scanning, but developed headaches within two days of scanning. To investigate whether the migraine phase affects the structural connectome organization, we conducted separate analyses for patients in the interictal, peri-ictal, and ictal phases.

#### D) Effects of depression and anxiety

To evaluate the impact of anxiety or depression on migraines, we assessed the differences in manifold eccentricity and degree values of subcortico-cortical structural connectivity between healthy controls and migraine patients without depression (*n* = 39), as well as between healthy controls and migraine patients with depression (*n* = 8). We further compared two between-group differences by calculating the linear correlation, and conducted the same analysis for patients with migraine without anxiety (*n* = 39) and with anxiety (*n* = 8).

#### E) Low- vs. high-frequency episodic migraine

A sensitivity analysis between low- and high-frequency episodic migraines was not conducted because of the small sample size of patients with high-frequency episodic migraines (*n* = 4). Instead, we repeated the analysis to assess the between-group differences using healthy controls and patients with migraines by considering only low-frequency episodic migraines (*n* = 43).

#### F) Manifold eccentricity calculation using multiple eigenvectors

We generated the manifold eccentricity using three eigenvectors that explained approximately 38% of the connectome information in the primary analysis. To assess the effects of the number of eigenvectors, we further performed independent analyses by calculating manifold eccentricity using multiple eigenvectors that explained approximately 50, 70, and 90% of the connectome information.

#### G) Between-group differences without controlling for age and sex

In the primary analysis, we controlled for age and sex, while assessing between-group differences between patients with migraines and healthy controls. In addition, we performed the analysis without controlling for age and sex.

## Results

### Study participants

We recruited 108 participants, including 65 patients with migraines and 43 healthy controls, and excluded 18 patients and two healthy controls. Specifically, we excluded seven patients who did not undergo T1-weighted or diffusion MRI scanning, as well as 11 patients and two healthy controls in whom cortical surface reconstruction failed due to severe head movements. Finally, 47 patients with migraine (mean ± SD age = 34.3 ± 8.3 years; sex = 74.5% female) and 41 healthy controls (age = 35.2 ± 7.7 years; sex = 75.6% female) were enrolled in this study. Among the patients with migraine, 26 were in the interictal phase, six in the peri-ictal phase, and 15 in the ictal phase. Seven patients had migraine with aura, while the remaining 40 did not. Patients showed a mean of 6.9 ± 4.84 days of headache per month and 4.2 ± 2.96 days of migraine per month. In particular, four patients had high-frequency episodic migraines (8 or more migraine days/month), eight had depression, and eight had anxiety. The detailed demographic and clinical information of the participants are presented in Table 1.

**Table 1:** Demographic and clinical information of study participants

Information	Patients (*N* = 47)	Control (*N* = 41)
Age (years)	34.3 (8.3)	35.2 (7.7)
Sex (male: female)	12:35	10:31
Disease duration (years)	12.1 (8.52)	N/A
Phase		
Interictal	26 (55.3%)	N/A
Peri-ictal	6 (12.8%)	N/A
Ictal	15 (31.9%)	N/A
Migraine subtype		
MOA	40 (85.1%)	N/A
MWA	7 (14.9%)	N/A
Monthly headache days	6.9 (4.84)	N/A
Monthly migraine days	4.2 (2.96)	N/A
HFEM (≥ 8 days/month of migraine)	4 (8.5%)	N/A
Depression (PHQ-9 scores of 8 or higher)	8 (17.0%)	0 (0%)
Anxiety (GAD-7 scores of 6 or higher)	8 (17.0%)	0 (0%)

Values are presented as the mean (standard deviation) or number (percentage)

*Abbreviations*: MOA, migraine without aura; MWA, migraine with aura; PHQ, Patient Health Questionnaire; GAD, Generalized Anxiety Disorder; N/A, not available

### Between-group differences in the manifold eccentricity

**Fig. 1:**
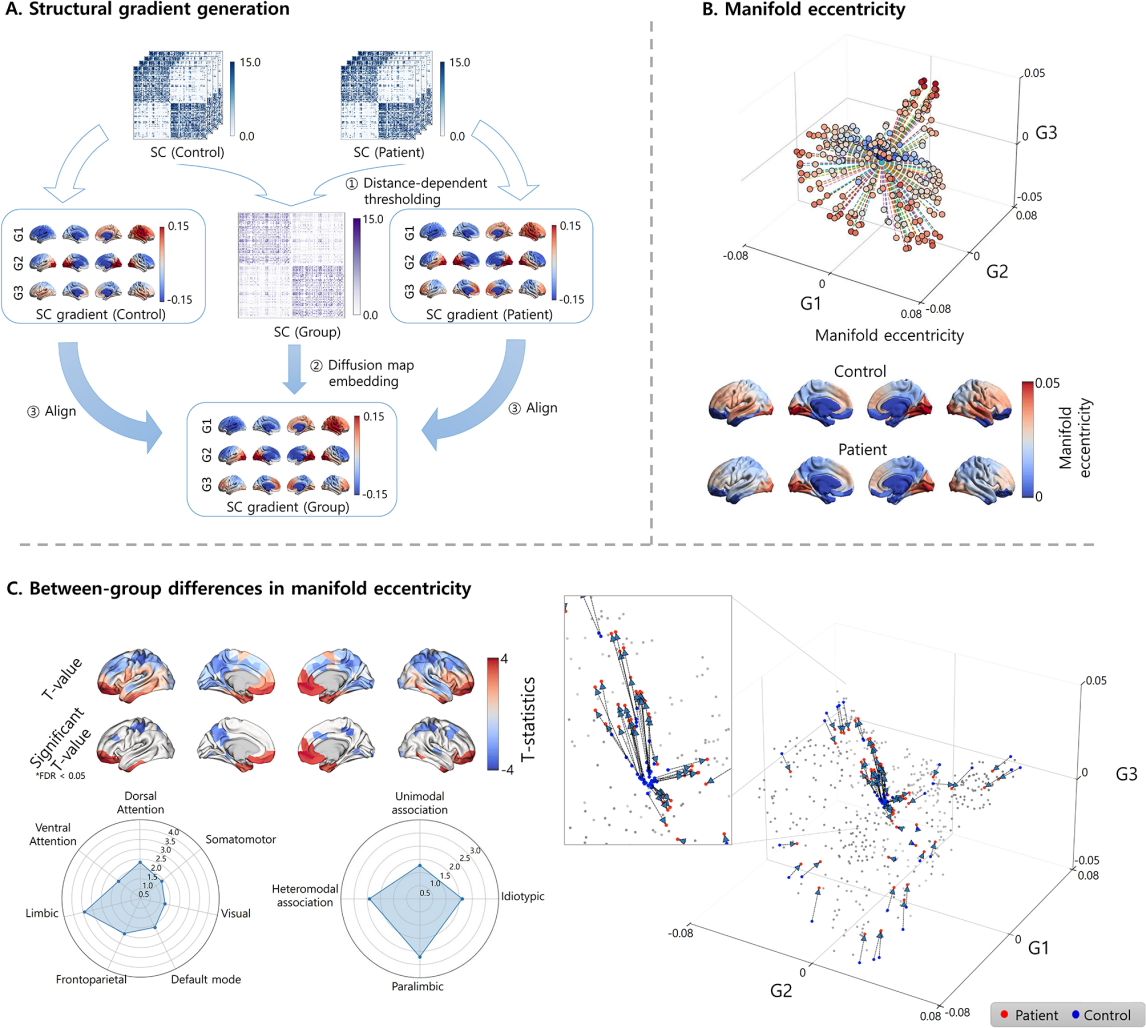
**Between-group differences in manifold eccentricity between patients with migraines and healthy controls.** **(A)** The structural connectome was estimated using diffusion MRI tractography *(top)*. ① The group representative matrix was computed using distance-dependent thresholding, and ② the group-level gradients (G1, G2, and G3) were generated using a diffusion map embedding algorithm. ③ Individual gradients were aligned to the group gradients using Procrustes alignment. **(B)** The schema of manifold eccentricity is shown with a three-dimensional scatter plot *(top)*. The average manifold eccentricity maps of healthy controls and patients with migraines are shown on the brain surfaces *(bottom).* **(C)** T-statistics of between-group differences in the manifold eccentricity are shown *(left top)*. The effects were stratified according to seven intrinsic function communities and four cortical hierarchical levels using spider plots *(left bottom)*. Expansion or contraction in the manifold eccentricity of each brain region is shown as dots using arrows to differentiate healthy controls *(blue)* from patients with migraines *(red)*. Gray dots indicate the regions that did not show significant effects *(right). Abbreviations*: FDR, false discovery rate; MRI, magnetic resonance imaging; SC, structural connectivity

We used the first three gradients that accounted for approximately 38.23% of the structural connectivity matrix. Consistent with previous studies [15, 35, 43], the first gradient (G1) showed left-right, the second (G2) demonstrated anterior-posterior, and the third (G3) exhibited sensory-transmodal axes (Fig. 1A). After calculating the manifold eccentricity (Fig. 1B) [15], we assessed between-group differences, which revealed significant expansions in the orbitofrontal cortex and temporal pole and contractions in the sensorimotor regions in patients with migraines (Fig. 1C). When summarizing the between-group differences according to the seven intrinsic functional communities [38] and cortical hierarchical levels [39], the limbic network showed the strongest effects, followed by the heteromodal association areas (Fig. 1C).

### Between-group differences in subcortico-cortical connectivity

**Fig. 2:**
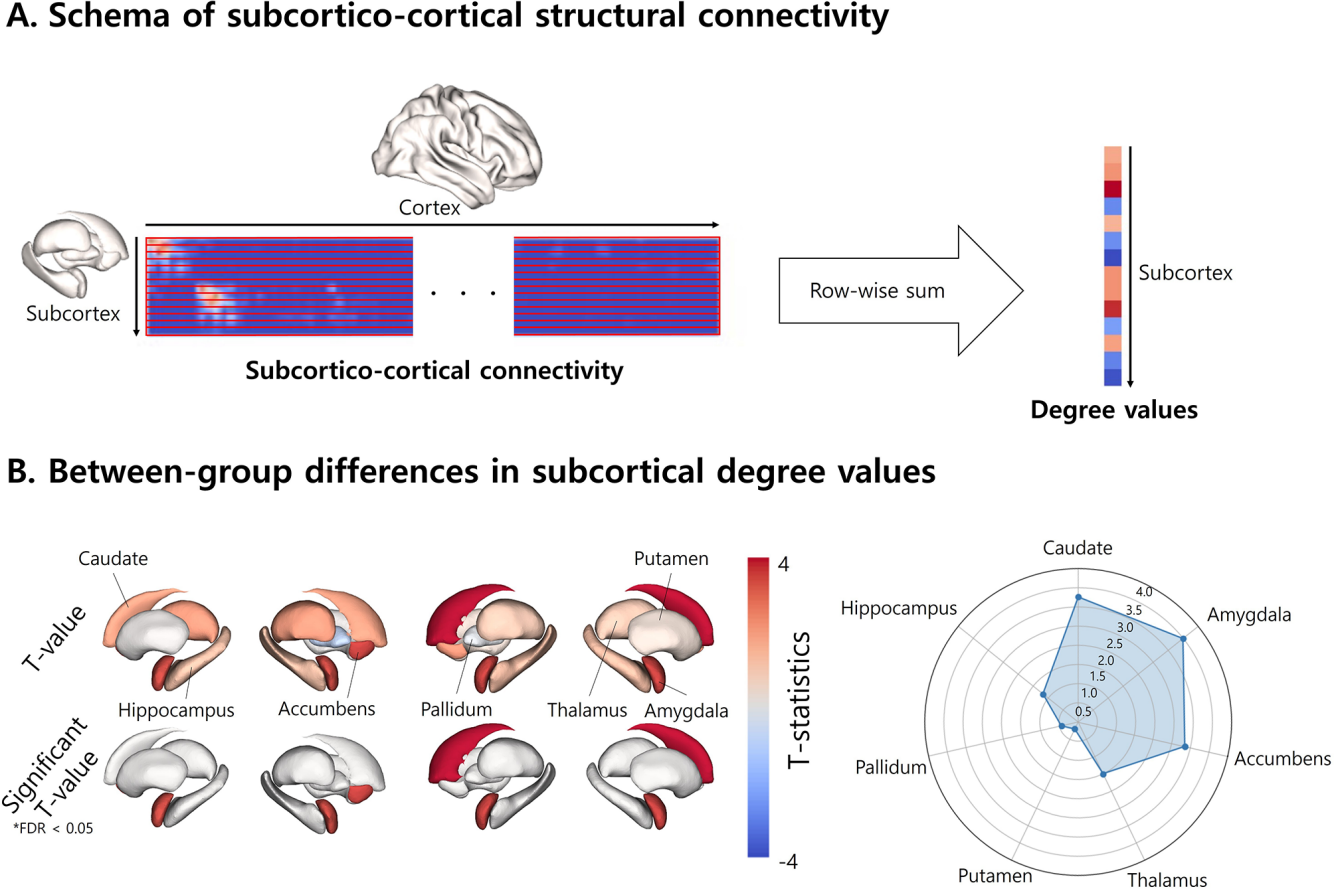
**Between-group differences in the subcortico-cortical structural connectivity.** **(A)** The degree values of each subcortical region were calculated with a row-wise sum of the subcortico-cortical structural connectivity. **(B)** The t-statistics of between-group differences in the degree values are reported on brain surfaces *(left)*, while the effects of each subcortical region are summarized using a spider plot *(right)*. *Abbreviation*: FDR, false discovery rate

Using the degree values of the subcortico-cortical structural connectivity (Fig. 2A), we identified significant between-group differences in the caudate, amygdala, and accumbens, characterized by increased degree values in patients with migraines (Fig. 2B).

### Classification between patients with migraine and healthy controls

**Fig. 3:**
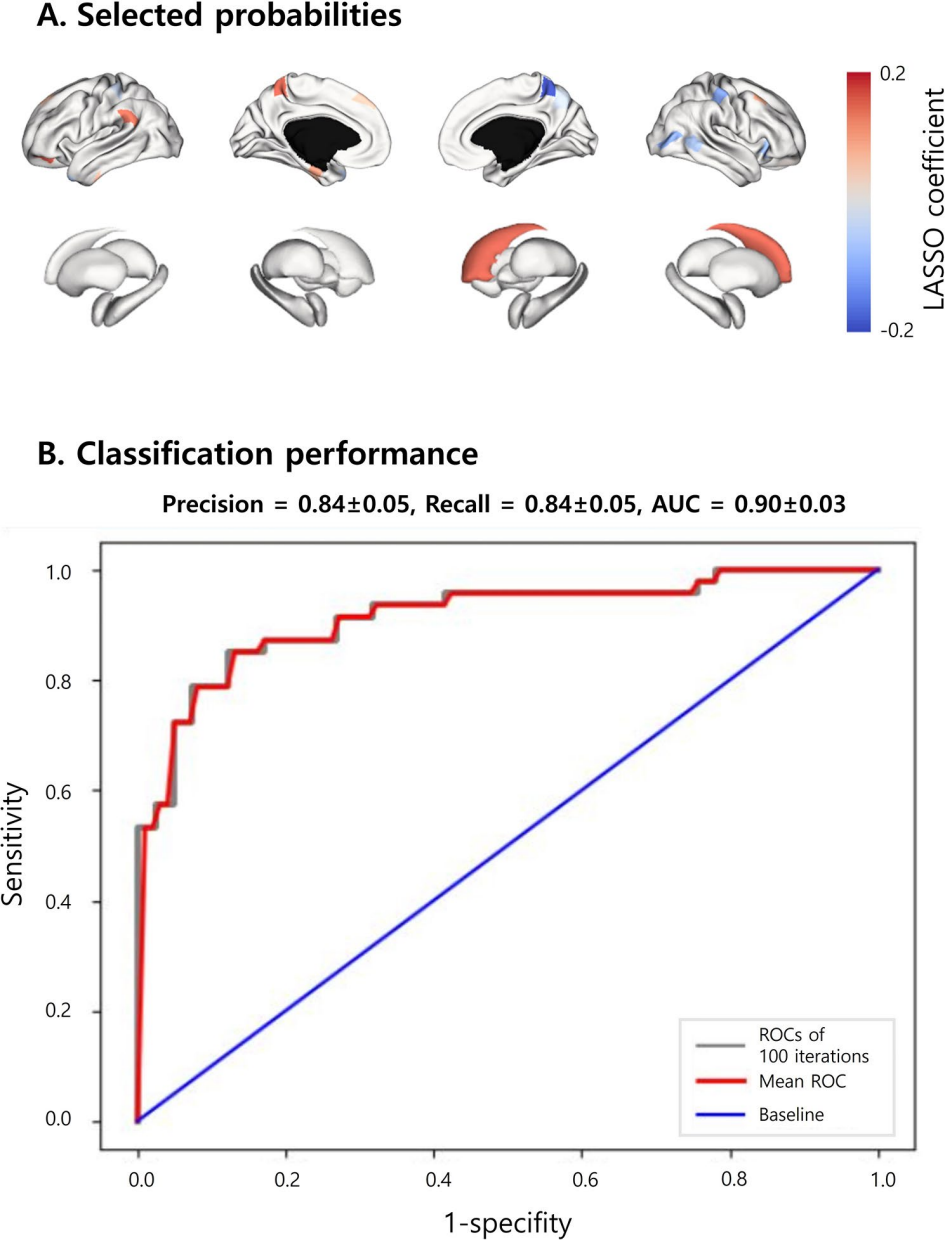
**Classification between patients with migraines and healthy controls.** **(A)** Selected probabilities based on the LASSO coefficient are shown on brain surfaces. **(B)** Prediction performance is shown as ROC curves. The red, gray, and blue lines indicate the mean ROC curve across 100 iterations, the ROC curves of all iterations, and the baseline, respectively. *Abbreviations*: AUC, area under the ROC curve; ROC, receiver operating characteristic; LASSO, least absolute shrinkage and selection operator

Across 100 repetitions, the sensory/motor cortex, inferior parietal lobule, temporal cortex, and caudate largely contributed to the group classification (Fig. 3A). The mean ± SD values for precision, recall, and AUC were 0.84 ± 0.05, 0.84 ± 0.05, and 0.90 ± 0.03, respectively (Fig. 3B), indicating that these structural connectivity features could be considered as markers for migraine diagnosis.

### Sensitivity analyses

Multiple sensitivity analyses demonstrated the robustness of our findings, as summarized below:

#### A) Excluding patients with migraine with aura

We repeated the analysis, excluding patients with migraine with aura, and observed that the spatial patterns of the t-statistics aligned with the main findings (linear correlation coefficient *r* = 0.92, *p* < 0.001; Supplementary Fig. 1).

#### B) Different parcellations

We further repeated the analysis using different spatial granularities based on the sub-parcellation of the Desikan–Killiany atlas with 200 and 400 parcels, and observed similar results (*r* = 0.78, *p* < 0.001 for 200 parcels; *r* = 0.91, *p* < 0.001 for 400 parcels; Supplementary Fig. 2A–B). We also assessed between-group differences using the Schaefer atlas with 300 parcels, which yielded consistent findings (*r* = 0.76, *p* < 0.001; Supplementary Fig. 2C). Overall, the results demonstrate the robustness of the findings across different parcellation schemes.

#### C) Different migraine phases

We examined the between-group differences in manifold eccentricity and degree values of subcortico-cortical structural connectivity in patients with migraines in the interictal, peri-ictal, or ictal phases, finding consistent results across different migraine phases: interictal (*r* = 0.90, *p* < 0.001), ictal (*r* = 0.92, *p* < 0.001), and peri-ictal (*r* = 0.52, *p* < 0.001; Supplementary Fig. 3).

#### D) Effects of depression and anxiety

We found consistent findings when comparing the statistics of migraine patients with depression or anxiety with those without depression or anxiety (*r* = 0.83, *p* < 0.001 for depression; *r* = 0.72, *p* < 0.001 for anxiety; Supplementary Fig. 4).

#### E) Low- vs. high-frequency episodic migraine

We assessed the between-group differences by considering only patients with low-frequency episodic migraines, and observed findings consistent with the main results (*r* = 0.93, *p* < 0.001; Supplementary Fig. 5).

#### F) Manifold eccentricity calculation using multiple eigenvectors

We repeated the assessment of between-group differences in manifold eccentricity using four, seven, and 11 eigenvectors that showed approximately 50%, 70%, and 90% of the explanations, respectively. These findings were largely consistent with the main results derived using the three eigenvectors (*r* = 0.95, *p* < 0.001 for four eigenvectors; *r* = 0.94, *p* < 0.001 for seven eigenvectors; and *r* = 0.91, *p* < 0.001 for 11 eigenvectors; Supplementary Fig. 6). These results indicate that these three eigenvectors may contain sufficient information to explain the connectome data.

#### G) Between-group differences without controlling for age and sex

When we assessed the between-group differences without controlling for age and sex, we observed similar findings to the initial analysis (*r* = 1.00, *p* < 0.001; Supplementary Fig. 7), indicating that age and sex did not significantly affect the results.

## Discussion

Migraines are a form of headache characterized by atypical brain structures; however, little is known about the structural connectome organization in patients with episodic migraines. Using manifold learning techniques, in the present study, we identified atypical connectome organization in the somatomotor and limbic networks in patients with migraines. We further demonstrated the utility of manifold features in distinguishing patients with migraines from healthy controls. Our findings provide insights into the understanding of macroscale structural connectome organization in patients with episodic migraines, further shedding light on the neural underpinnings of this condition.

Manifold learning is an approach used to delineate brain organization. In the present study, we applied this approach to structural connectivity data to define structural manifold eccentricity. Previous functional MRI studies have revealed a distinct cortical hierarchy along the sensory-transmodal axis [8, 10]. Similarly, another study based on microstructural profiles revealed a similar pattern known as the sensory-fugal axis [44]. In the present study, we applied this approach to structural connectivity data and defined structural manifold eccentricity. The manifold eccentricity depicts the distance between each brain region and the center of all data points in the manifold space, thereby quantifying changes in connectome organization in terms of expansion and contraction [15]. Prior research has shown that the expansion of manifold eccentricity within the manifold space may be interpreted as increases in nodal and within-module connectivity and segregation with other brain regions [15]. In the present study, we found that the limbic regions, particularly the orbitofrontal cortex and temporal pole, showed expansion of the connectome manifold. These findings may indicate greater differentiation between connectivity with limbic regions compared to connectivity with the rest of the brain. In contrast, the sensorimotor regions exhibited contractions in manifold eccentricity, indicating more integrated patterns in the brain.

The orbitofrontal cortex is involved in pain-related brain networks that mediate changes in the value of pain [45], in which altered serotonergic and dopaminergic systems may disrupt sensory integration and decision-making processes [46, 47]. The temporal pole participates in pain processing by mediating affective responses to stimuli [48–50]. In the present study, we found that these limbic cortices showed an expansion of manifold eccentricity in patients with migraines, suggesting more dissimilar connectome patterns compared with other brain regions. Our findings further indicate that expansions in manifold eccentricity in the limbic cortex are related to a larger differentiation in connectivity from other brain regions, which could lead to reduced efficiency in processing sensory and cognitive information. Sensory processing abnormalities have been well described in patients with migraines [51–55]. In particular, the pain matrix, which consists of the thalamic, sensorimotor, and prefrontal regions, integrates pain-related sensory and cognitive responses, and contributes to active nociceptive processing [56, 57]. Although the exact biological underpinnings of the pain matrix remain debatable, it is clear that altered nodal connectivity in sensory regions is associated with pain processing in patients with migraine.

Subcortical regions are also crucial for understanding the brain of patients with migraines. For example, the amygdala receives sensory inputs from the limbic cortices of the orbitofrontal, temporal, and cingulate regions, delivering this information to subcortical structures [58]. These subcortico-cortical circuits play a role in pain modulation, while alterations in these circuits are known to increase pain [58]. In one previous study, patients with episodic migraines showed that altered connectivity in the reward system, specifically between the accumbens and amygdala, might have been affected by endogenous dopaminergic signaling, which could be the underlying neural mechanism for pain processing in patients with migraines [59]. As such, increased degree values in the subcortico-cortical connectivity in the accumbens and amygdala may indicate increased reception of pain information from the cortical areas.

Multiple sensitivity analyses considering different spatial scales, types of parcellation scheme, the number of eigenvectors used to construct manifold eccentricity, and removal of potential confounding effects by considering migraine with/without aura and ictal/peri-ictal/interictal phases have demonstrated the reliability and robustness of our findings. We found consistent between-group differences across different migraine phases, indicating the role of the structural connectome as a reliable diagnostic marker for migraine. Furthermore, a machine learning framework for classifying patients with migraines and healthy controls validated the clinical utility of our approach. Although the generalizability of the findings should be tested using independent external datasets in future studies, the present results enhance our understanding of the macroscale perspectives of the brains of migraine patients.

In the present study, we employed manifold learning techniques to assess the changes in the structural connectome organization using diffusion MRI-based tractography data. We thus provide insights into the structural connectivity in patients with migraines, extending beyond the functional alterations explored in our previous study [14]. This study contributes to a deeper understanding of whole-brain structural connectivity in patients with migraines, potentially facilitating the discovery of robust neuroimaging biomarkers.

This study has some limitations which should be considered. First, we did not assess the effect of the white matter hyperintensity, which has been shown to be associated with migraine pathology [60, 61]. This parameter can be measured using fluid-attenuated inversion recovery data, and future studies are required to investigate the relationship between white matter hyperintensity burden and structural connectome changes. Second, our analysis focused on identifying structural connectome changes in patients with migraines and their implications for group classification. In future studies, we will further explore how these changes are related to headaches. Third, several methodological choices can be considered for constructing structural connectivity; these may include: tractography approaches, either deterministic or probabilistic; filtering methods, including spherical deconvolution informed filtering of tractograms [28, 62] and convex optimization modeling for microstructure informed tractography [63, 64]; and quantification of structural connectivity, such as fiber count, density, or cross-section streamlines. Fourth, we only studied the structural connectivity changes in patients with migraines in the current study. Further investigation of the interactions between the structural and functional networks may provide new insights into migraine connectopathy. Finally, the current study explored connectivity changes in patients with episodic migraine. Comparisons of connectome changes between patients with episodic and chronic migraines, as well as the effects of psychological disorders, should be investigated further.

### Electronic supplementary material

Below is the link to the electronic supplementary material.

Supplementary Material 1

## Data Availability

All data supporting the findings of this study will be made available upon request from the corresponding author, Mi Ji Lee. The data are not publicly available due to Institutional Review Board restrictions. The codes for eigenvector generation are available at https://github.com/MICA-MNI/BrainSpace; the codes for calculating network communication measures are available at https://sites.google.com/site/bctnet/; and the codes for statistical analyses are available at https://github.com/MICA-MNI/ENIGMA.
