# Supplementary material for: Shifts in structural connectome organization in the limbic and sensory systems of patients with episodic migraine
Source: J Headache Pain. 2024 Jun 11;25(1):99. doi: 10.1186/s10194-024-01806-2 (PMC11165833; doi:10.1186/s10194-024-01806-2)
Supplement: Supplementary file 1 — Supplementary Material 1 [file 10194_2024_1806_MOESM1_ESM.docx]

**Supplementary information**


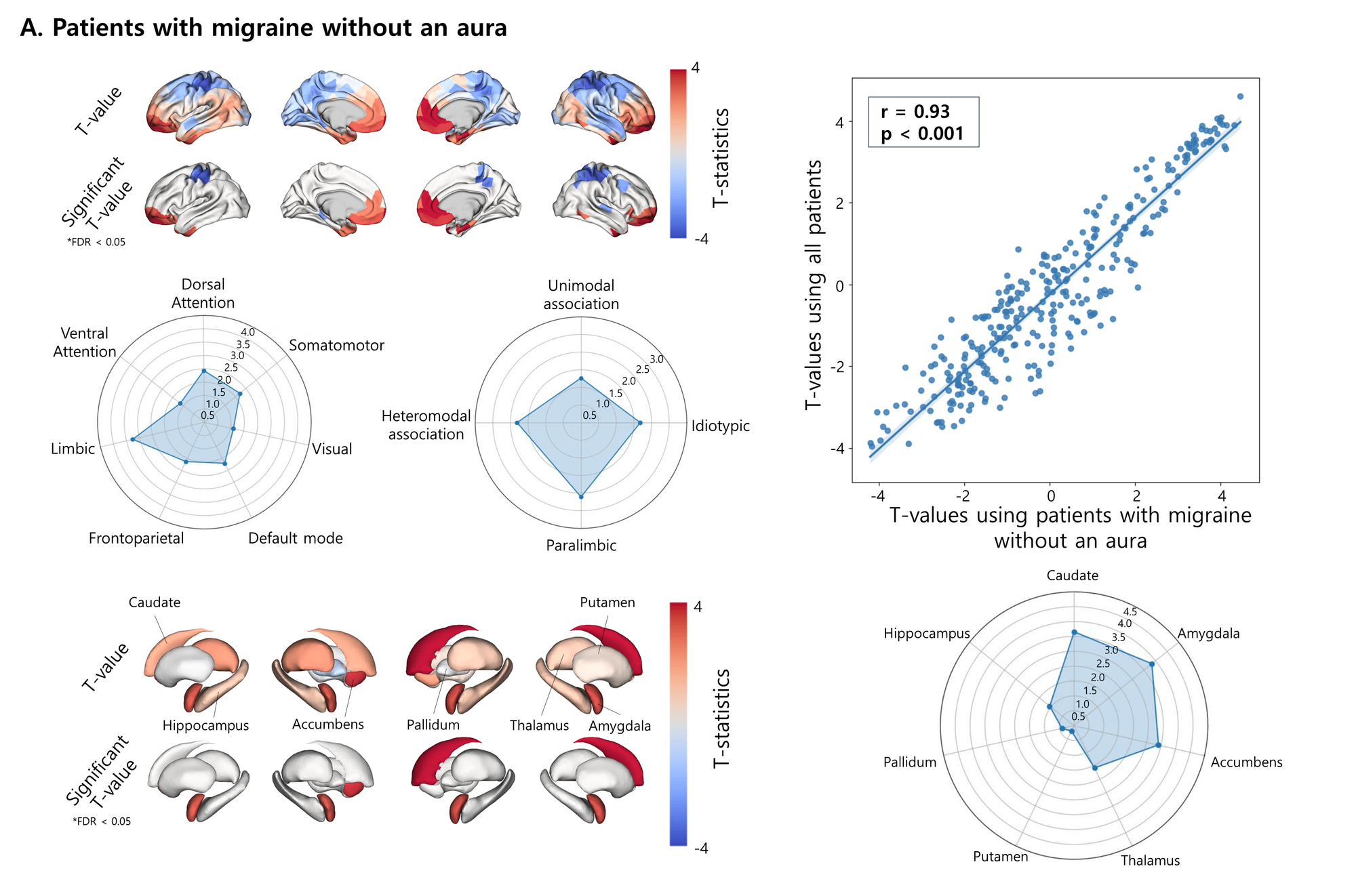


**Supplementary Fig. 1.** **Between-group differences in structural connectivity between patients with migraine without an aura and healthy controls.** T-statistics of the between-group differences in the manifold eccentricity are reported on brain surfaces *(left top)*. The effects are stratified according to seven intrinsic function communities and four cortical hierarchical levels, and reported using spider plots *(left middle)*. The expansion or contraction in manifold eccentricity of each brain region are shown as dots using arrows emanating from healthy controls (blue) to patients with migraine (red). Gray dots indicate the regions that did not show significant effects *(right top)*. The t-statistics of between-group differences in the degree values are reported on brain surfaces *(left bottom)*. The effects of each subcortical region are summarized using a spider plot *(right bottom)*.

*Abbreviation:* FDR, false discovery rate.


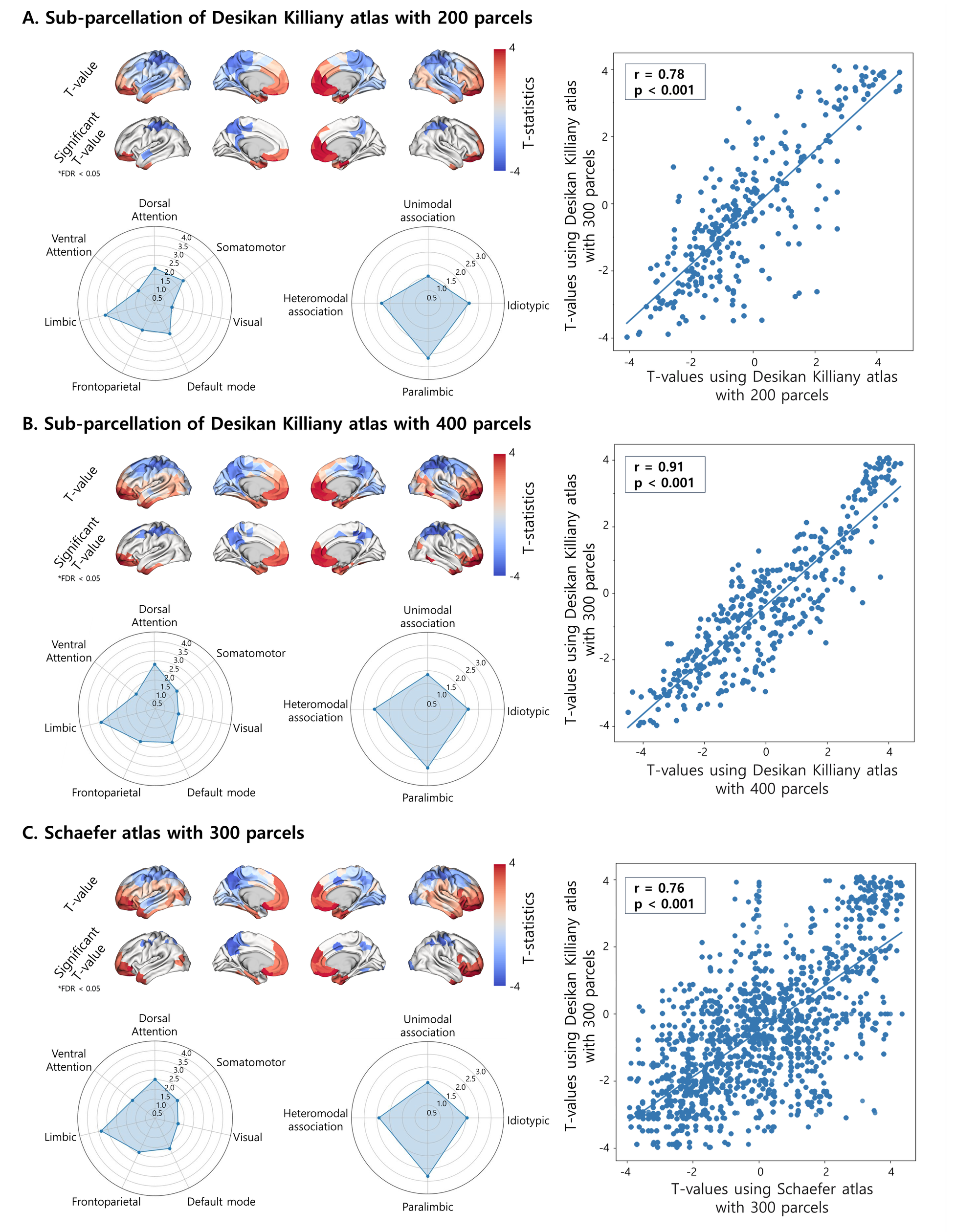


**Supplementary Fig. 2. Between-group differences in manifold eccentricity using different parcellations schemes. (A)** The results are shown based on the sub-parcellation of Desikan–Killiany atlas with 200 parcels, **(B)** 400 parcels, and **(C)** Schaefer atlas with 300 parcels. For details, see *Supplementary Fig. 1*.

*Abbreviation:* FDR, false discovery rate.


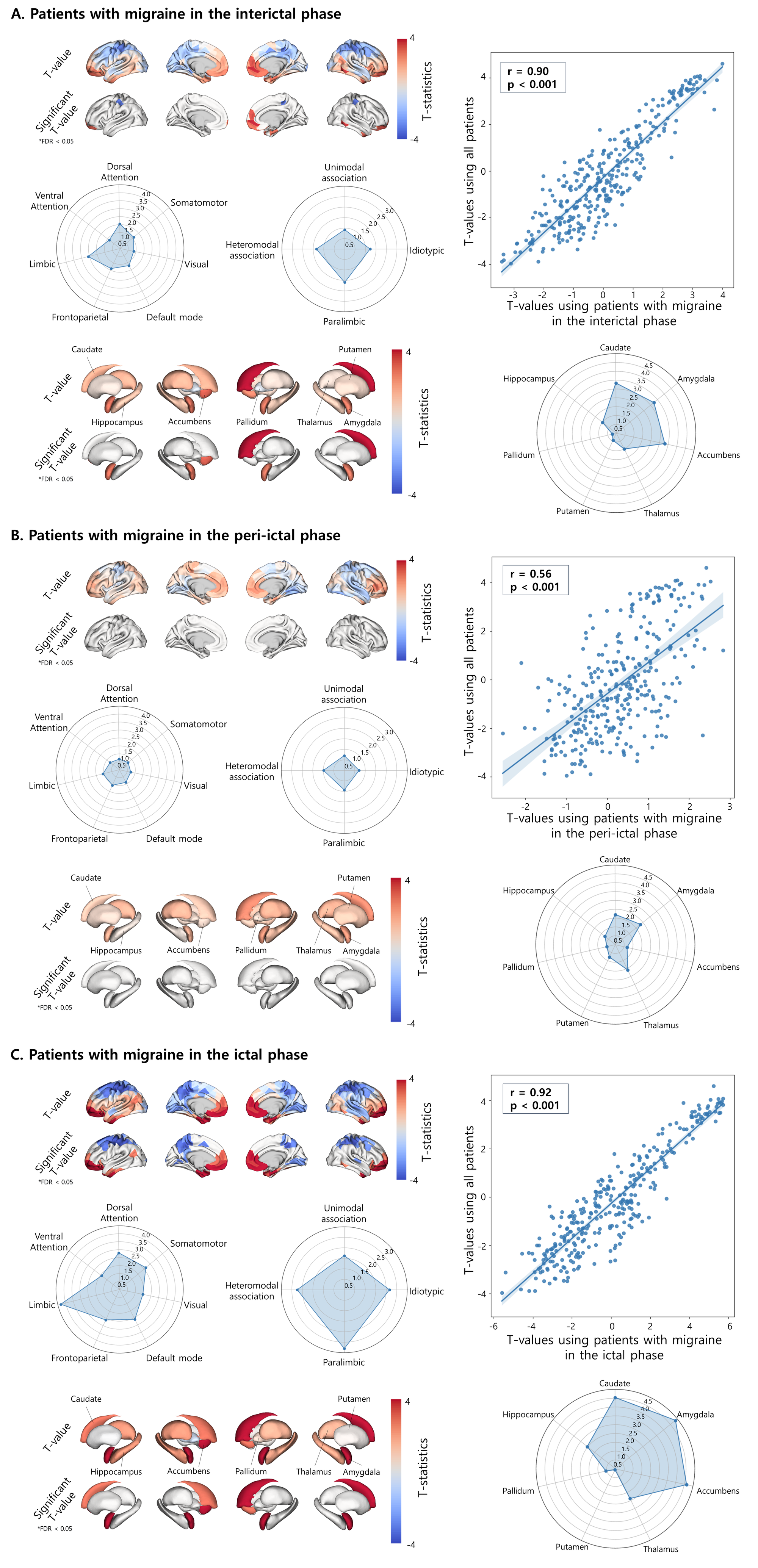


**Supplementary Fig. 3.** **Between-group differences in structural connectivity using patients with migraine at different phases**. **(A)** The results based on patients with migraine in the interictal phase, **(B)** the peri-ictal phase, **(C)** and the ictal phase are shown. For details, please see *Supplementary Fig. 1*.

*Abbreviation:* FDR, false discovery rate.

*
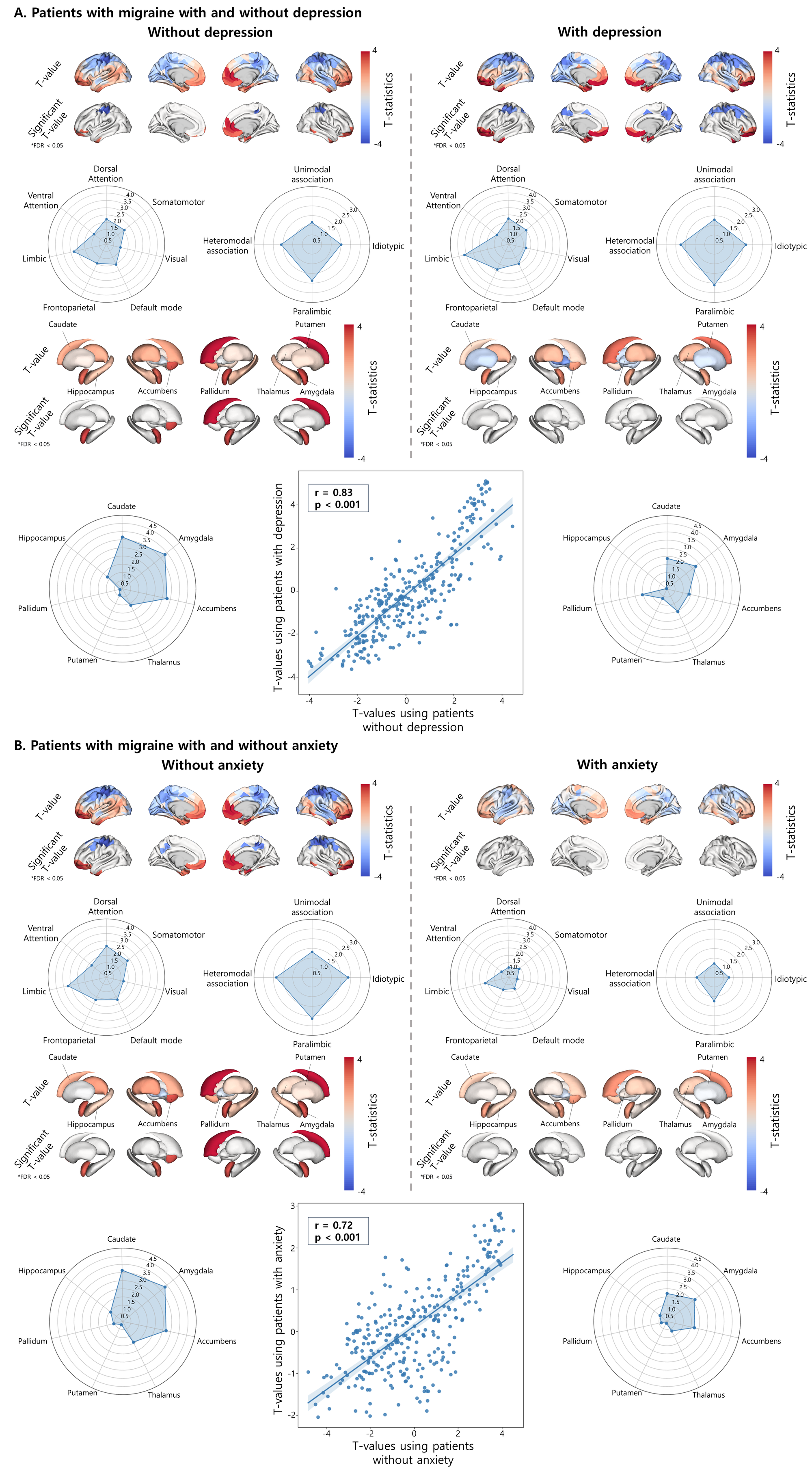
*

**Supplementary Fig. 4. Between-group differences in structural connectivity using patients with and without depression or anxiety.** For details, please see *Supplementary Fig. 1*.

*Abbreviation:* FDR, false discovery rate.


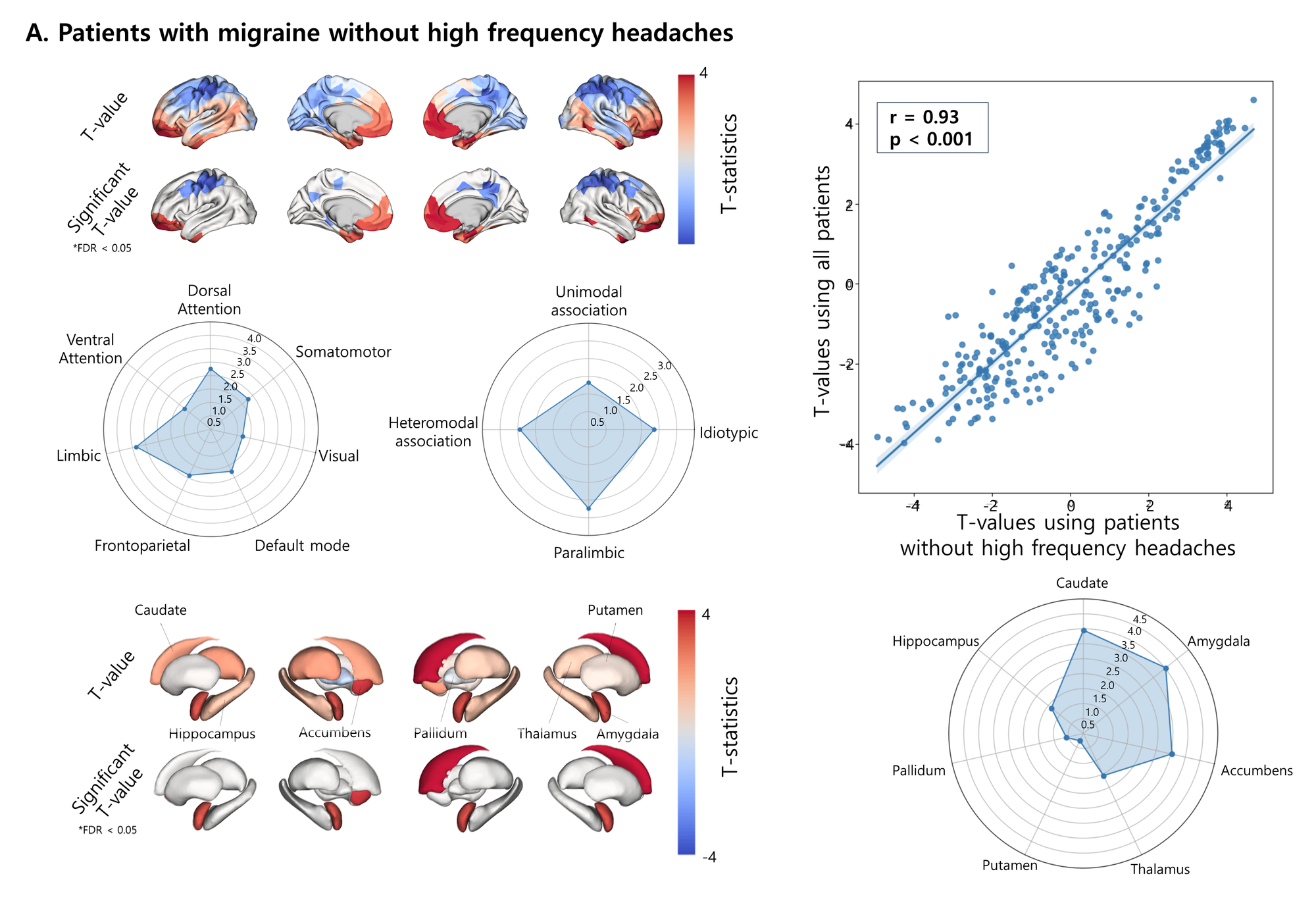


**Supplementary Fig. 5. Between-group differences in structural connectivity using healthy controls and patients with migraine, without considering high-frequency episodic migraine.** For details, please see *Supplementary Fig. 1*.

*Abbreviation:* FDR, false discovery rate.


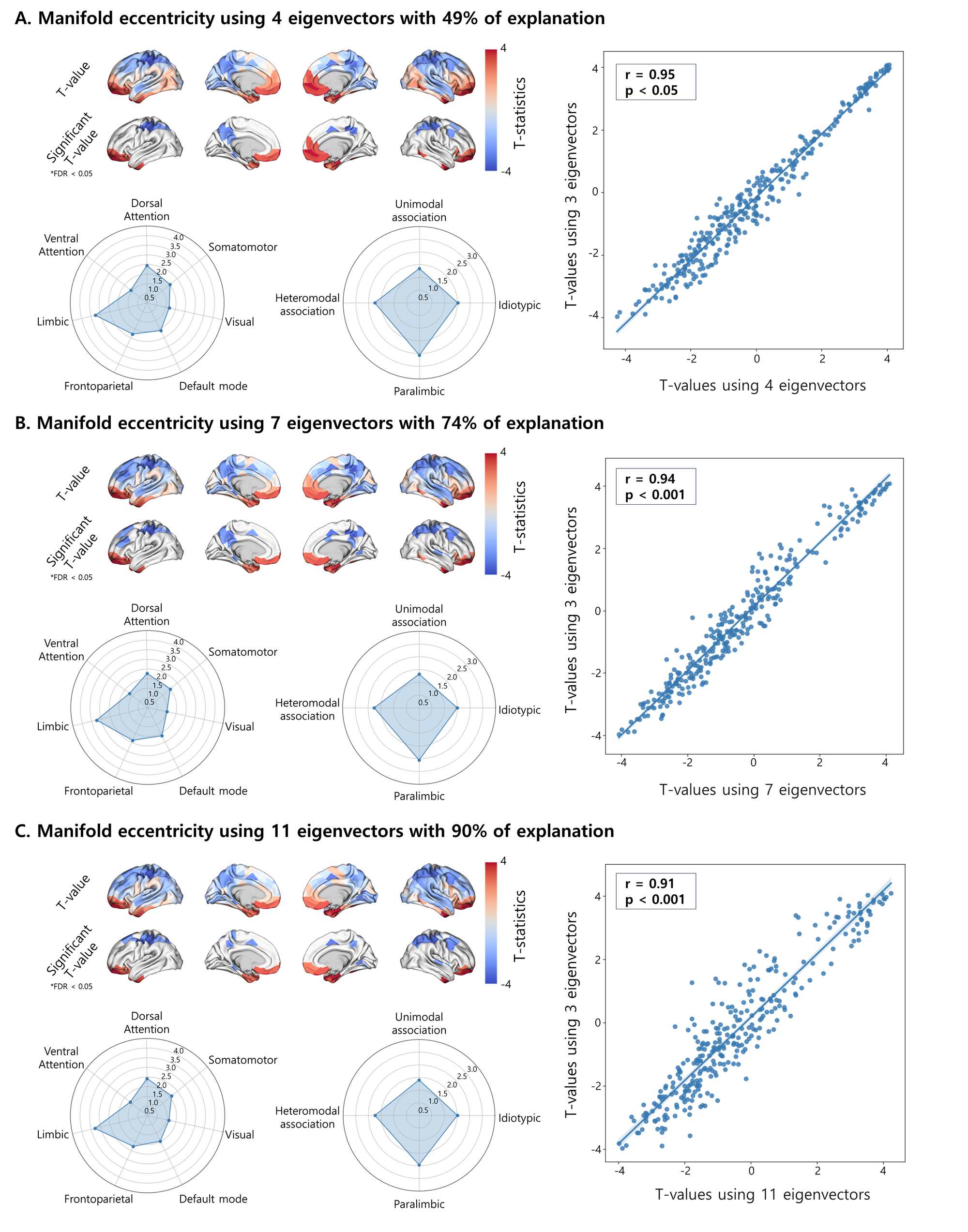


**Supplementary Fig. 6. Between-group differences in manifold eccentricity using multiple eigenvectors. (A)** The results based on the manifold eccentricity using four, **(B)** seven, and **(C)** 11 eigenvectors are shown. For details, please see *Supplementary Fig. 1*.

*Abbreviation:* FDR, false discovery rate.


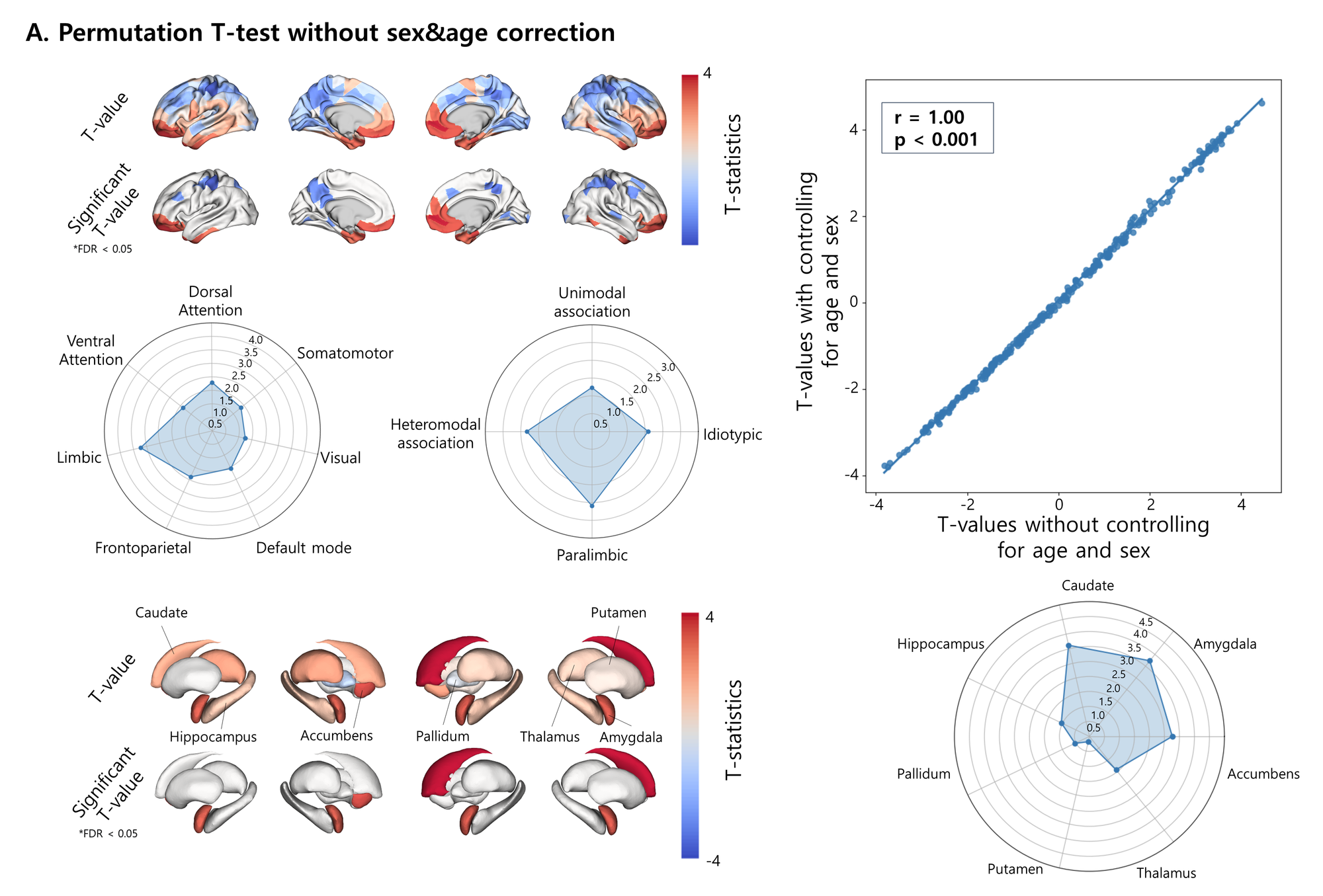


**Supplementary Fig. 7. Between-group differences in the structural connectivity without controlling for age and sex.** For details, please see *Supplementary Fig. 1*.

*Abbreviation:* FDR, false discovery rate.
